# Identification and Validation of the miR/RAS/RUNX2 Autophagy Regulatory Network in AngII-Induced Hypertensive Nephropathy in MPC5 Cells Treated with Hydrogen Sulfide Donors

**DOI:** 10.3390/antiox13080958

**Published:** 2024-08-07

**Authors:** Qing Ye, Mi Ren, Di Fan, Yicheng Mao, Yi-Zhun Zhu

**Affiliations:** 1 Shanghai Key Laboratory of Bioactive Small Molecules, School of Pharmacy, Fudan University, Shanghai 201203, China; 2 The Department of Hepatobiliary Surgery and Liver Transplantation, Shanghai General Hospital, Shanghai Jiaotong University School of Medicine, Shanghai 200080, China; 3 State Key Laboratory of Quality Research in Chinese Medicines, (R & D Center) Lab. for Drug Discovery from Natural Resource, School of Pharmacy, Macau University of Science and Technology, Macau 999078, China

**Keywords:** hypertensive nephropathy, hydrogen sulfide donors, miRNA, autophagy, bioinformatics analysis

## Abstract

The balanced crosstalk between miRNAs and autophagy is essential in hypertensive nephropathy. Hydrogen sulfide donors have been reported to attenuate renal injury, but the mechanism is unclear. We aimed to identify and verify the miRNAs and autophagy regulatory networks in hypertensive nephropathy treated with hydrogen sulfide donors through bioinformatics analysis and experimental verification. From the miRNA dataset, autophagy was considerably enriched in mice kidney after angiotensin II (AngII) and combined hydrogen sulfide treatment (H_2_S_AngII), among which there were 109 differentially expressed miRNAs (DEMs) and 21 hub ADEGs (autophagy-related differentially expressed genes) in the AngII group and 70 DEMs and 13 ADEGs in the H_2_S_AngII group. A miRNA–mRNA–transcription factors (TFs) autophagy regulatory network was then constructed and verified in human hypertensive nephropathy samples and podocyte models. In the network, two DEMs (miR-98-5p, miR-669b-5p), some hub ADEGs (*KRAS*, *NRAS*), and one TF (*RUNX2*) were altered, accompanied by a reduction in autophagy flux. However, significant recovery occurred after treatment with endogenous or exogenous H_2_S donors, as well as an overexpression of miR-98-5p and miR-669b-5p. The miR/RAS/RUNX2 autophagy network driven by H_2_S donors was related to hypertensive nephropathy. H_2_S donors or miRNAs increased autophagic flux and reduced renal cell injury, which could be a potentially effective medical therapy.

## 1. Introduction

Hypertension is recognized as one of the leading causes linked to the development of cardiovascular and renal disease [1,2], and it also promotes the progression of chronic kidney disease towards end-stage renal disease [3]. Hypertensive nephropathy is mainly manifested as renal structural and functional damage caused by essential hypertension, among which renal inflammation is an important pathological feature [4,5]. In essential hypertension, the renin–angiotensin–aldosterone system (RAAS) is extensively activated, and the expression of its main effector angiotensin II (AngII) is upregulated. These subsequently lead to the dysfunction of renal endothelial cells, the release of various inflammatory factors, and the proliferation of mesangial cells, thereby inducing vascular remodeling, promoting renal inflammation and fibrosis, and ultimately leading to a decreased glomerular filtration rate, glomerular ischemia, sclerosis, and nephron atrophy [6,7]. Therefore, AngII is an important mediator of hypertensive nephropathy, and it is also the modeling condition chosen by many studies [8]. In addition, many studies have shown that damage to and structural changes in podocytes are very important in renal injury [9]. Here, we employed AngII-induced podocytes as a cell model. Autophagy is an important and highly conserved pathway, which acts as an essential role in maintaining the homeostasis of physiological and pathological processes, such as immunity, inflammation, aging and metabolism, etc. [10]. Accumulating evidence suggests that hypertensive nephropathy may be associated with the imbalance of autophagy regulation, and targeting the autophagy pathway may alleviate hypertension-induced kidney damage [9,11].

Hydrogen sulfide (H_2_S) is an important physiological gas molecule, and it along with nitric oxide (NO) and carbon monoxide (CO) are considered as the three major gas signaling molecules in the body [12]. Endogenous hydrogen sulfide is synthesized by cystathionine-γ-lyase (CSE), cystathionine-β-synthase (CBS), and 3-mercaptopiruvate sulfurtransferase (MST) enzymes [13]. Previous studies have shown that hydrogen sulfide is involved in the regulation of various physiological and pathological conditions, including the cardiovascular, renal, respiratory, central nervous, and digestive systems [14]. In common kidney diseases, such as CKD and acute kidney injury, hydrogen sulfide production is reduced, and the expression of CBS, CSE, or MST is also downregulated to varying degrees [15,16,17]. Treatment with hydrogen sulfide donors not only restores hydrogen sulfide levels but also ameliorates kidney function [18]. Multiple studies have shown that hydrogen sulfide donors alleviate renal damage by regulating autophagy, oxidative stress, inflammation, and cell injury through various signaling pathways [19]. However, few studies have focused on the epigenetic regulation of hydrogen sulfide donors in the treatment of hypertensive nephropathy, especially on microRNA regulation.

MicroRNAs (miRNAs, miRs) are a group of single-stranded non-coding RNAs with a length of 19–22 nucleotides, which are important regulators of gene post-transcriptional expression [20]. By specifically binding to the 3′UTR sequences of mRNA, miRNAs lead to the degradation or post-transcriptional inhibition of target genes and then participate in the regulation of different biological processes [21]. The dysregulation of miRNAs is important in the occurrence and development of various kidney diseases [22]. In hypertensive nephropathy, many miRNAs, such as miR-200a, miR-200b, miR-141, miR-429, miR-205 and miR-192, have increased expression [23]. Studies have shown that angiotensin-converting enzyme inhibitors downregulate the expression of miR-324-3p in glomeruli and tubules, thereby reducing renal fibrosis [24]. However, few studies have addressed miRNAs regulating autophagy pathways in hypertensive nephropathy. In addition, a growing number of studies have shown that hydrogen sulfide can also regulate miRNAs to improve cardiac and renal dysfunction [25,26]. Since the role of miRNAs in hypertensive nephropathy is emerging, the functional miRNAs and corresponding key mRNAs that regulate the autophagy pathway in hypertensive nephropathy and hydrogen sulfide donor therapy are explored to construct the miRNA-mRNA regulatory network, which is helpful for elucidating the molecular mechanism of hydrogen sulfide donors in the treatment of hypertensive nephropathy.

Herein, we screened out differentially expressed miRNAs (DEMs) after AngII or a combination of AngII and hydrogen sulfide treatment using the Gene Expression Omnibus (GEO) database. Then, their target mRNAs were predicted by the validation and prediction databases, the top 5% hub mRNAs were identified using the protein–protein interaction (PPI) network and CytoHubba, and potential autophagy-related differentially expressed genes (ADEGs) were identified by the autophagy-related gene set. Subsequently, functional annotation and pathway enrichment analysis were conducted and a miRNA-ADEGs-TF (transcription factor) regulatory network was constructed and validated to better understand the underlying mechanism of the hydrogen sulfide treatment of hypertensive nephropathy.

## 2. Materials and Methods

### 2.1. Data Acquisition

**Figure 1:**
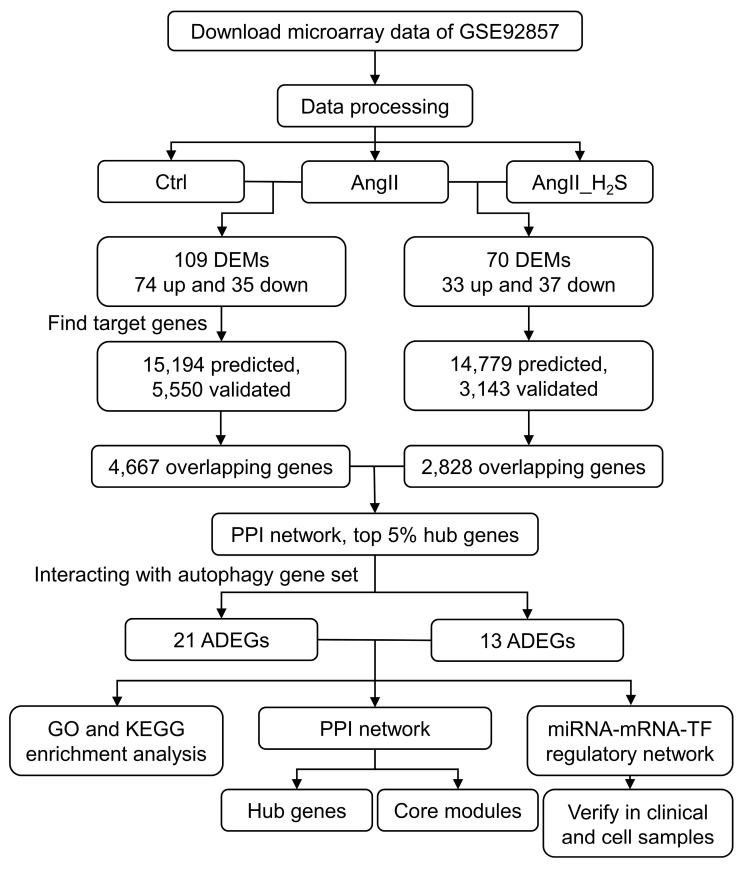
The flowchart of screening strategy. Ctrl, control kidney at 28 days. AngII, mice treated with AngII via mini osmotic pump for 28 days. AngII_H_2_S, mice treated with AngII via mini osmotic pump and hydrogen sulfide donor via i.p. injection for 28 days. DEMs, differentially expressed miRNAs. ADEGs, potential autophagy differentially expressed genes. GO, Gene Ontology. KEGG, Kyoto Encyclopedia of Genes and Genomes. PPI, protein–protein interaction. TF, transcription factor.

In order to obtain genetic analysis data for hypertensive nephropathy and hydrogen sulfide treatment, we used “hypertension”, “kidney”, and “hydrogen sulfide” as keywords to search for genome-wide expression studies in the GEO database (https://www.ncbi.nlm.nih.gov/geo/, accessed on 1 October 2022). The non-coding RNA expression profiling dataset GSE92857 was screened out and downloaded using the R package “GEO query” (v 2.62.2). The GSE92857 dataset, containing 12 kidney tissues each from normal control (Ctrl), AngII-treated (AngII), hydrogen-sulfide-treated, and a combination of AngII and hydrogen sulfide (AngII_H_2_S)-treated mice, was compiled on the GPL19117 ([miRNA-4] Affymetrix Multispecies miRNA-4 Array) platform [26]. This dataset was normalized with the RMA (robust multiarray analysis) algorithm using the Partek Genomics Suite software (Partek v6.6, St. Louis, MO, USA), and the independent GSE37455 and GSE37460 datasets containing human hypertensive nephropathy samples were utilized for validation. The workflow chart of the methodology used in this study is shown in Figure 1.

### 2.2. Analysis of Differentially Expressed miRNAs

The normalized data matrix of the miRNA profiling dataset was acquired and represented as boxplot and density plot. The probes in the dataset were then annotated with the annotation file from the GPL19117 platform, and only probes interrogating M. Muculus transcripts were included. We screened out DEMs comparing the AngII and Ctrl groups and the AngII_H_2_S and AngII groups using the “limma” package (v 3.50.3) of the R software (v 4.1.2) [27]. MiRNAs with a *p* value less than 0.05 and an absolute fold change (FC) greater than 1.5 were considered as DEMs. Box line, density, and volcano plots of DEMs were generated using the “ggplot2” package (v 3.3.6) [28], and the circular heatmap of the DEMs was illustrate through the TBtools software (v 1.098769).

### 2.3. Prediction and Identification of Hub Target Genes

The multiMiR package is a database of miRNAs and target genes, disease, and drug interactions, including 8 prediction databases (DIANA-microT-CDS, ElMMo, MicroCosm, miRanda, miRDB, PicTar, PITA, and TargetScan), 3 experimentally validated databases (miRecords, miRTarBase, and TarBase), and 3 databases containing drug-/disease-related information (miR2Disease, Pharmaco-miR, and PhenomiR) [29]. The predicted and validated target genes of the DEMs were selected by multiMiR package (v 2.3) analysis, and the intersection of the two sets was selected as the target genes of DEMs. The PPI network of these intersecting target genes was then constructed using the Cytoscape software (v 3.9.1), followed by the Cytohubba (v 0.1) plug-in’s Degree algorithm to take the top 5% genes as hub target genes [30,31].

### 2.4. Selection of Autophagy-Related Genes

The autophagy-related gene set in Mus musculus was collected from the entries mmu04140 and mmu04136 in the Kyoto Encyclopedia of Genes and Genomes (KEGG) pathway database (https://www.genome.jp/entry/mmu04140, https://www.genome.jp/entry/mmu04136, accessed on 1 November 2022). The autophagy-related gene set is provided in Table S1. In addition, we identified potential ADEGs via the Venn diagram package (v 1.7.3).

### 2.5. Functional and Pathway Enrichment Analysis of ADEGs

To evaluate the biological functions of the DEMs targeting ADEGs, Gene Ontology (GO) enrichment analysis and KEGG pathway analysis were performed using the “ClusterProfiler” package (v 4.2.2) in the R software. Among them, the species was set to Mus musculus, and the Benjamini–Hochberg method was used to adjust the *p* value. Finally, the enrichments or pathways with adjusted *p* values of less than 0.05 were screened out for analysis and plot display.

### 2.6. PPI Network and Recognition of Hub ADEGs and Core Modules

The PPI network of ADEGs was visualized using the STRING database (https://string-db.org/, v 11.5, accessed on 8 November 2022), with the interaction score setting at a high confidence (0.700) [32]. Afterward, the analysis data of the PPI network was imported into the Cytoscape software (v 3.9.1), and the CytoHubba plug-in (v 0.1) was used to assign values to each gene through the topological network algorithm, and then the top 10 genes of the “Degree” attribute were regarded as hub ADEGs [31]. In addition, the Molecular Complex Detection (MCODE, v 2.0.2) plug-in was utilized to analyze the core modules [33].

### 2.7. Construction of miRNA-mRNA-TF Regulatory Network

Based on the results of the screened DEMs and targeted ADEGs, an autophagy-related miRNA-mRNA regulatory network was constructed using the Cytoscape software (v 3.9.1). Transcription factors (TFs) and miRNAs are two important regulators of gene expression, often controlling gene regulatory networks in a dependent or independent manner [34,35]. In order to further study the molecular mechanism of hydrogen sulfide regulation of autophagy, we used the iRegulon plug-in (v 1.3) to predict potential transcription factors associated with ADEGs [36]. Ultimately, transcription factors enriched in the top 5 fractions, miRNAs co-regulated by AngII and hydrogen sulfide, and corresponding ADEGs were selected to construct an autophagy-related miRNA-mRNA-TF regulatory network.

### 2.8. Drugs and Reagents

S-Propargyl-cysteine (SPRC), a substrate donor for H_2_S synthesis, was synthesized and purified as described in our previous study [37,38]. NaHS, interferon-γ, DL-Propargylglycine (PAG), and angiotensin II (AngII) were purchased form Sigma-Aldrich (St. Louis, MO, USA). Losartan was obtained from MCE (Shanghai, China). RPMI 1640 medium, fetal bovine serum (FBS), penicillin/streptomycin solution, and 0.25% trypsin were purchased from Gibco (New York, NY, USA). RNAiso Plus and RNAiso for small RNA reagents were obtained from TaKaRa (Shiga, Japan). A Hifair III 1st Strand cDNA Synthesis Kit (gDNA digester plus) and Hieff miRNA Universal qPCR SYBR Master Mix were obtained from Yeasen(Shanghai, China). ABScript III RT Master Mix for qPCR and 2X Universal SYBR Green Fast qPCR Mix were obtained from ABclonal (Wuhan, China). The miR-98-5p mimic (micrON mmu-miR-98-5p mimic), miR-669b-5p (micrON mmu-miR-669b-5p mimic), miR-NC (micrON mimic NC), and ribo*FECT*™ CP were obtained from RiboBio (Guangzhou, China).

### 2.9. Cell Culture

Conditionally immortalized mouse podocyte MPC5 cells were purchased from ATCC (USA). The MPC5 cells were cultured and differentiated as previously described [39]. The MPC5 cells were first cultured at 33 °C in RPMI 1640 medium containing 10% FBS, 1% penicillin/streptomycin, and 30 U/mL recombinant mouse interferon-γ. Then, the MPC5 cells were placed at 37 °C and cultured in complete RPMI 1640 medium without interferon for 10–14 days, and further experiments were performed after differentiation and maturity. When the MPC5 cells reached 80% confluence, they were digested with 0.25% trypsin and passaged into new culture dishes at a ratio of 1:3 to continue culturing.

For miRNAs mimic transfection, MPC5 cells were seeded into a 6-well plate containing complete culture medium the day before transfection, allowing the cell density to be about 40% at the time of transfection. Then, according to the manufacturer’s instructions, the transfection mixture was prepared and added dropwise to the cells in complete medium without penicillin/streptomycin, and the culture was continued in a 37 °C incubator for 48 h for subsequent AngII treatment and other operations.

For the H_2_S donor treatment, MPC5 cells were treated in the following groupings: Ctrl group (vehicle), AngII group (1 μM AngII treatment), NaHS or SPRC group (pre-treated with 50 μM NaHS or 50 μM SPRC for 1 h before exposure to 1 μM AngII), and SPRC + PAG group (pre-incubated with 2 mM PAG for 30 min and then pre-treated with 50 μM SPRC for 1 h before exposure to 1 μM AngII). For quantitative PCR and WB, AngII was stimulated for 24 or 72 h, respectively. NaHS, SPRC, and PAG were freshly prepared before each experiment.

### 2.10. Quantitative PCR

The total RNA and miRNA were extracted using RNAiso Plus and RNAiso for small RNA reagents according to the manufacturer’s protocol. For the mRNA targets, total RNA was reversed into cDNA using ABScript III RT Master Mix. Subsequent quantitative PCR (qPCR) experiments were performed in a Bio-Rad CFX96 PCR instrument using 2X Universal SYBR Green Fast qPCR Mix. For miRNAs, complementary DNA (cDNA) was synthesized using specific stem–loop primers for miR-98-5p and miR-669b-5p through a Hifair III 1st Strand cDNA Synthesis Kit. Then, qPCR analyses were run in triplicate to calculate miRNAs expression using Hieff miRNA Universal qPCR SYBR Master Mix. U6 snRNA and Gapdh were used as a reference for miRNAs and mRNAs. All the primers used are shown in Table S2.

### 2.11. Western Blot

Western blot (WB) analysis was performed as previously reported [40]. Briefly, proteins in MPC5 cells were lysed and extracted with RIPA buffer (Yeasen, China) and then boiled in 5× loading buffer (Yeasen, China). Subsequently, the protein concentration in each sample was determined using a BCA kit (Yeasen, China). Samples with the same amount of protein were subjected to SDS-PAGE (sodium dodecyl sulfate–polyacrylamide gel electrophoresis) and transferred to polyvinylidene fluoride (PVDF, Millipore, Burlington, MA, USA) membranes. The membranes were then blocked with 5% nonfat dry milk (Cell Signaling, Danvers, MA, USA) and incubated with the following primary antibodies: anti-PTEN, anti-KRAS, anti-NRAS, anti-RUNX2, anti-CSE, anti-MST (1:1000, Abclonal, Wuhan, China), anti-MAPK1 (Erk1/2), anti-LC3B (1:1000, Cell Signaling, Danvers, MA, USA), anti-P62 (SQSTM1, 1:5000), anti-nephrin, anti-podocin, anti-CBS (1:1000, Proteintech, Rosemont, IL, USA), and anti-GAPDH (1:10,000, ABclonal, Wuhan, China). After washing with 1X TBST (Tris-buffered saline with tween 20), secondary HRP goat anti-rabbit or anti-mouse antibodies (1:10,000, Abclonal, Wuhan, China) combined with immobilon Western chemiluminescent HRP substrate (Millipore) were used to visualize bands under a ChemiDoc XRS+ system (BIO-RAD, Hercules, CA, USA). The grey value of each band was calculated using the Image J software (v1.54f, NIH, Bethesda, MA, USA), and the relative expression of the target protein was determined using the internal reference protein GADPH.

### 2.12. H_2_S Content Assay

H_2_S content was measured as per the manufacturer’s instructions (H_2_S content assay kit, Solarbio, Beijing, China). In brief, a certain number of cells were collected using the extraction solution, and they were then subjected to ultrasound and centrifugation. The reaction reagents were added and incubated at room temperature for 10 min. Subsequently, the absorbance of the solution at 665 nm was detected using a microplate reader (Varioskan Flash, Thermo Scientific, Waltham, MA, USA), and the corresponding H_2_S concentration was obtained by substituting it into the standard curve of the standard solution concentration.

For fluorescence detection, a WSP-1 fluorescent probe (MCE, Shanghai, China) for H_2_S was used. After stimulation, 10 μM WSP-1 was added and incubated at 37 degrees for 60 min. After a quick wash with PBS twice, 10 μg/mL Hoechst 33342 staining solution (Yeasen, Shanghai, China) was added and incubated for 10 min. Finally, observations and recordings were made under an Olympus CKX53 inverted fluorescence microscope (Tokyo, Japan).

### 2.13. Statistics

In addition to the above-mentioned R package, the GraphPad Prism 9 (v. 9.0.0, Boston, MA, USA) software was used for graphing and statistical analysis. For pairwise comparisons between more than two group sets, one-way ANOVA was performed, and Tukey’s multiple comparison post hoc test was utilized to correct the *p* value. Statistical significance was considered when the resulting *p* value was less than 0.05. All experiments were repeated at least three times independently, and data are shown as mean ± SD.

## 3. Results

### 3.1. MiRNAs Were Dysregulated in Mouse Kidney after AngII Induction and H_2_S Donor Treatment

A dataset of miRNA expression in kidneys from AngII- and/or H_2_S-treated mice was downloaded from the GEO database. The distribution trend in the boxplot of this dataset was basically a straight line (Figure S1A), and the density plots of the values in the different groups were basically coincident (Figure S1B). The results showed that the data quality was good and had good repeatability.

**Figure 2:**
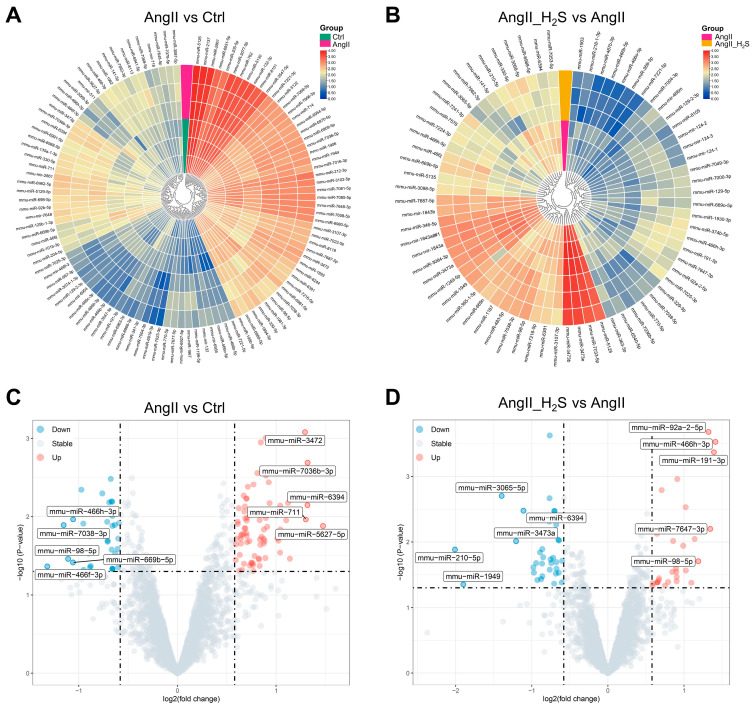
Screening of DEMs in AngII- or H_2_S-treated mouse kidney. Clustered heatmap of DEMs between AngII and Ctrl groups (**A**) as well as comparison of AngII and H_2_S donor treatments (AngII_H_2_S) and AngII alone group (**B**). Green label represents Ctrl group, fuchsia represents AngII group, and khaki represents AngII_H_2_S group. The miRNA expression values were processed by log base 2 and are represented from blue to red in ascending order. (**C**) The volcano plot of DEMs between AngII and Ctrl groups. (**D**) The volcano plot of DEMs between AngII_H_2_S and AngII groups. Each of the top five miRNAs with the largest difference in forward and reverse changes are marked. Down means downregulated miRNAs, and these are shown as blue dots. Up, presented as red dots, means upregulated miRNAs. The gray dots show the not significantly changed miRNAs. The criteria were absolute FC > 1.5 and *p* value < 0.05.

We revealed a total of 109 DEMs between the AngII and Ctrl groups, with 74 miRNAs upregulated and 35 miRNAs downregulated according to the principle of absolute FC > 1.5 and *p* value < 0.05 (Figure 2A,C). After the AngII and H_2_S donor treatment, a total of 70 DEMs were screened out in comparison with the AngII alone group, with 33 miRNAs upregulated and 37 miRNAs downregulated (Figure 2B,D). The hierarchical clustering analysis and volcano plots were used to assess the variation between the different groups (Figure 2). All the DEMs are listed in Supplementary Tables S3 and S4. These results showed the presence of abundant altered miRNAs in mouse kidneys after treatment with AngII alone or in combination with hydrogen sulfide donors.

### 3.2. The Target Genes of These Dysregulated miRNAs Were Mainly Concentrated in the Autophagy Pathway

**Figure 3:**
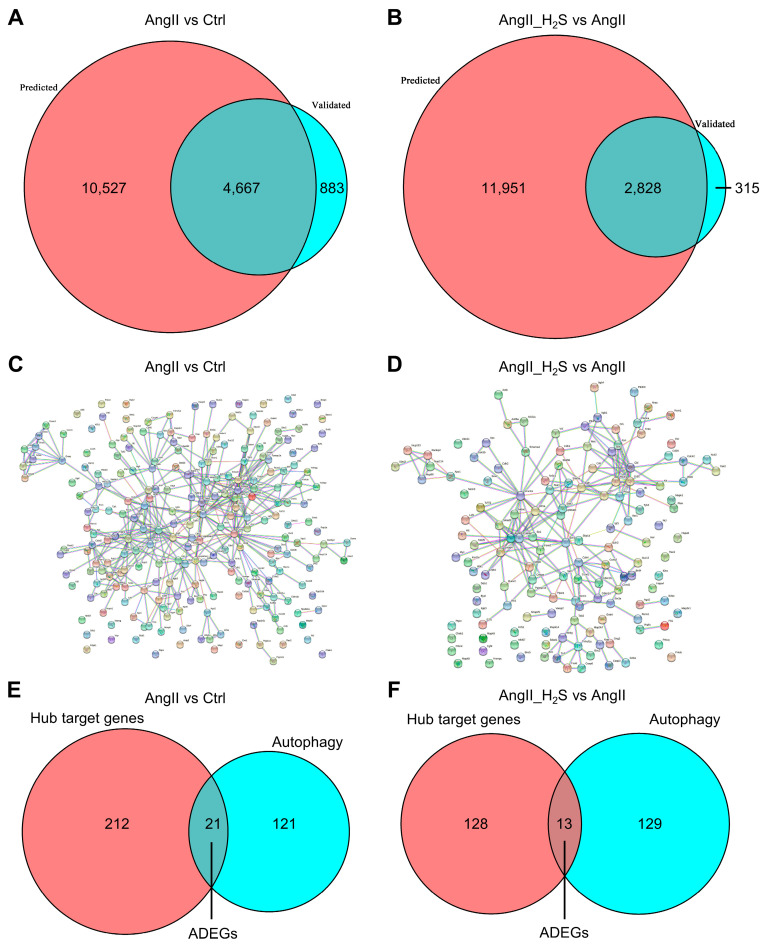
Hub target genes and ADEGs were selected for DEMs associated with AngII alone or AngII plus H_2_S donor treatment. Venn diagrams showing predicted target genes (red) and validated target genes (sky blue) corresponding to DEMs in comparison between AngII vs. Ctrl (**A**) and AngII_H_2_S vs. AngII (**B**). PPI network displaying the interaction of hub target genes from comparison between AngII vs. Ctrl (**C**), and AngII_H_2_S vs. AngII (**D**). The interacting genes between hub target genes and autophagy-related gene set in AngII vs. Ctrl (**E**) and AngII_H_2_S vs. AngII (**F**) comparison.

In order to identify the potential functions of these DEMs, we used eight prediction databases and three validation databases in the multiMiR database to find the target genes of these DEMs. In total, 5550 validated and 15,194 predicted target genes and 3143 validated and 14,779 predicated target genes were found in the AngII vs. Ctrl and AngII_H_2_S vs. AngII groups, respectively. Next, overlapping genes among the predicted and validated genes were selected as candidate target genes by crossover analysis. A total of 4667 and 2828 candidate target genes were selected accordingly (Figure 3A,B). To further screen hub target genes, candidate genes were imported into the STRING database and Cytoscape software, and the genes with the top 5% of the PPI scores were marked as hub target genes according to the Cytohubba plug-in. A total of 233 and 141 hub target genes were marked in the AngII vs. Ctrl and AngII_H_2_S vs. AngII groups, respectively, and these were displayed with the PPI network (Figure 3C,D). Afterwards, these hub target genes were interacted with the autophagy-related gene set to obtain 21 and 13 potential ADEGs, respectively (Figure 3E,F and Supplementary Table S5).

**Figure 4:**
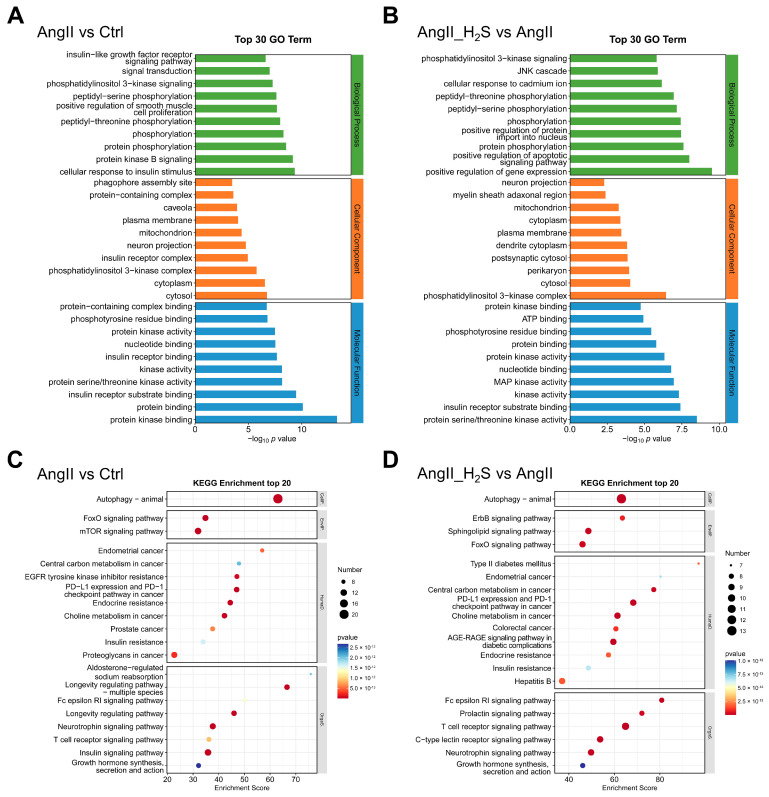
Enrichment plots from GO and KEGG functional analysis of targeted ADEGs. Barplots of the enrichment analysis for each of the top 10 biological processes (green), cellular component (orange-red), and molecular function (blue) from AngII vs. Ctrl (**A**) and AngII_H_2_S vs. AngII (**B**). In addition, the barplots are arranged from smallest to largest according to the negative logarithm of the adjusted *p* value. The KEGG pathway enrichment analysis results of AngII vs. Ctrl (**C**) and AngII_H_2_S vs. AngII (**D**) are shown as dotplots. Among them, the size of the dots represents the number of ADEGs hitting the corresponding pathway, and the adjusted *p* value of each pathway is displayed from red to blue in ascending order. CellP., cellular processes; EnvIP., environmental information processing; HumaD., human diseases; OrgaS., organismal systems.

In an effort to gain insight into the functions and mechanisms of these ADEGs, the GO and KEGG databases were used to enrich GO terms, including biological process (BP), cellular component (CC), and molecular function (MF), as well as the KEGG pathway via the ClusterProfiler package. In the comparison between the AngII and Ctrl groups, the enrichment analysis of the top 30 GO terms is shown in Figure 4A. In the biological process category, AngII mainly affected the phosphorylation and activation of proteins such as protein kinase B signaling, protein phosphorylation, and peptidyl-threonine phosphorylation the mouse kidney. In addition, in terms of cellular components, AngII mainly altered aspects such as the phosphatidylinositol 3-kinase complex and mitochondria, while the major molecular functions affected were protein binding and enzymatic activity (Figure 4A). In contrast, after treatment with hydrogen sulfide donors, protein phosphorylation in biological processes was also enriched, in addition to the positive regulation of gene expression and the JNK cascade (Figure 4B). In the cellular component and molecular function categories, hydrogen sulfide donor treatment also altered the phosphatidylinositol 3-kinase complex as well as protein binding and kinase activation (Figure 4B).

The enrichment results of the top 20 KEGG pathways of ADEGs from the two comparison groups are shown in Figure 4C,D. In the KEGG enrichment analysis, AngII mainly regulated autophagy, the mTOR signaling pathway, and the FoxO signaling pathway in kidney (Figure 4C). Similarly, the H_2_S donor treatment also modulated autophagy, the FoxO signaling pathway, and the ErbB signaling pathway (Figure 4D). These enriched GO terms and KEGG signaling pathways may be the main mechanism involved in the H_2_S treatment of hypertensive renal injury caused by AngII.

### 3.3. The Hub ADEGs of These Dysregulated miRNAs Were Analyzed

**Figure 5:**
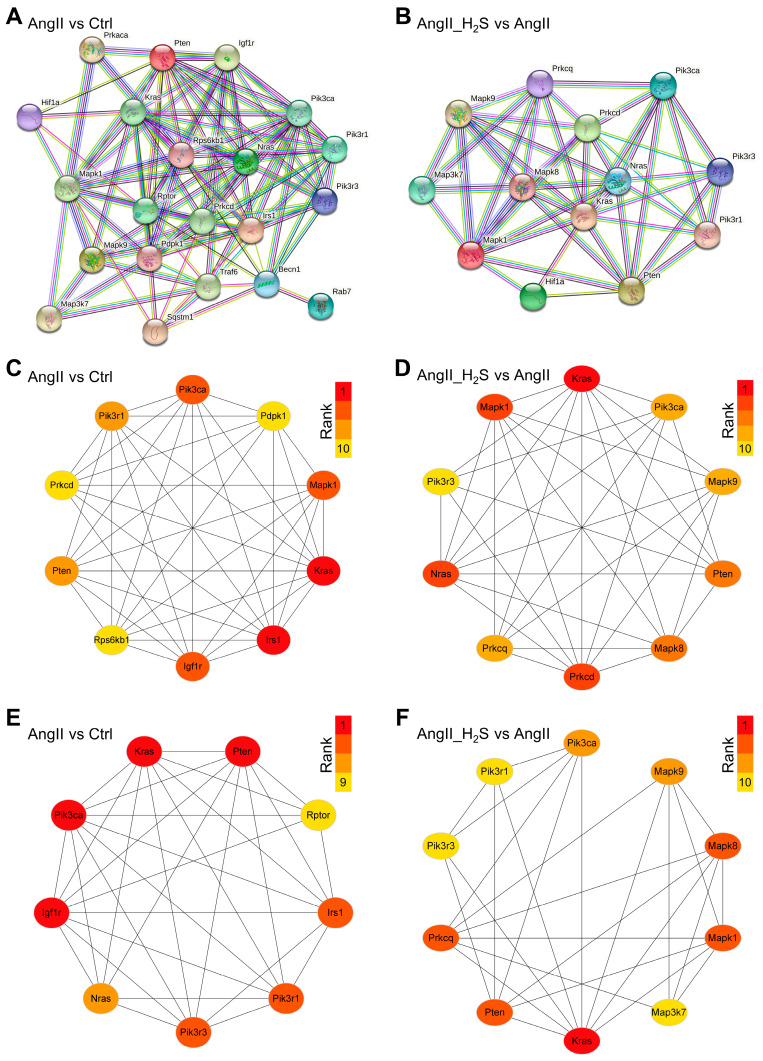
Protein–protein interaction networks and core modules of ADEGs constructed by STRING and Cytoscape. PPI analysis of targeted ADEGs from AngII vs. Ctrl (**A**) and AngII_H_2_S vs. AngII (**B**). The network illustration of top 10 hub ADEGs explored by CytoHubba in comparison between AngII vs. Ctrl (**C**) and AngII_H_2_S vs. AngII (**D**). Hub ADEGs are ranked by Degree value from dark red to orange red. Core modules analyzed by molecular complex detection network clustering analysis in AngII vs. Ctrl (**E**) and AngII_H_2_S vs. AngII (**F**).

To further investigate the function of the targeted ADEGs at the protein level and reveal the core interactions affected by hydrogen sulfide donor treatment, we constructed PPI networks from the Ctrl vs. AngII and AngII vs. AngII_H_2_S groups using the STRING database (Figure 5A,B). The PPI networks of Ctrl vs. AngII with 21 nodes and 196 interaction relationships and AngII vs. AngII_H_2_S with 13 nodes and 94 edges are shown in Figure 5A and B, respectively. Then, the top 10 hub ADEGs were determined based on the cytoHubba Degree algorithm using the Cytoscape software. After the AngII treatment, the hub ADEGs identified were Kras, Irs1, Igf1r, Pik3ca, Mapk1, Pten, Pik3r1, Rps6kb1, Pdpk1, and Prkcd. (Figure 5C). As for the H_2_S donor treatment, Kras, Nras, Mapk1, Prkcd, Mapk8, Pten, Pik3ca, Mapk9, Prkcq, and Pik3r3 were found to be hub ADEGs (Figure 5D). Meanwhile, the core modules in these PPI networks were analyzed using MCODE with the k-score set to 2.0. A sub-network consisting of 9 nodes and 32 edges was considered as a core cluster in Ctrl vs. AngII, and Pik3ca, Pten, Kras, and Igf1r occupied the center of the module (Figure 5E). In addition, the core module of AngII vs. AngII_H_2_S contained 10 nodes and 27 edges, and its center was Kras (Figure 5F). The hub ADEGs and core modules revealed by these results may be important mechanisms by which hydrogen sulfide donors and AngII regulate the renal autophagy network.

### 3.4. Construction of the miRNA-mRNA-TF Autophagy Regulatory Network for H_2_S Donor and/or AngII Treatment

**Figure 6:**
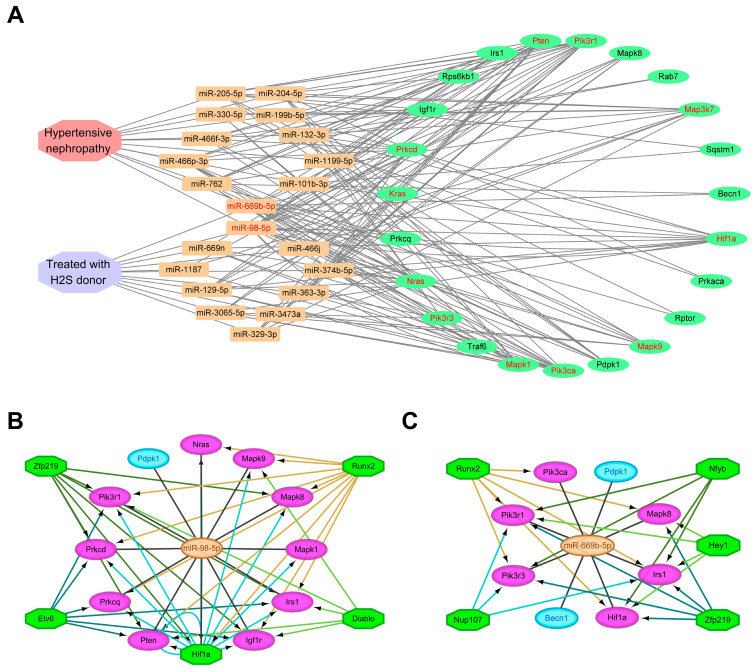
Regulatory networks of the DEMs, target hub ADEGs, and transcription factors. (**A**) The miRNA-mRNA autophagy regulatory network in AngII-induced hypertensive nephropathy and hydrogen sulfide donor drug therapy. Octagons, rectangles, and ovals represent diseases/drugs, DEMs, and target hub ADEGs, respectively. The red fonts represent miRNAs and mRNAs co-regulated by AngII and H_2_S donors. The miRNA-mRNA-TF autophagy regulatory network of miR-98-5p (**B**) and miR-669b-5p (**C**). Green octagons represent transcription factors, blue ovals indicate target genes regulated by miRNAs, and purple ovals show target genes regulated by both miRNAs and transcription factors.

Finally, the DEMs and corresponding potential hub ADEGs of the two comparison groups were summarized and visualized using Cytoscape. This miRNA-mRNA regulatory network consisted of two main factors, namely AngII-induced hypertensive nephropathy and hydrogen sulfide donor treatment, as well as 21 differentially expressed miRNAs and 23 key ADEGs (Figure 6A). To further elucidate the functions of these miRNAs and highlight the key processes of the transcriptional control of key ADEGs, regulatory networks of common miRNA–mRNA–transcription factors were constructed, respectively. The top five transcription factors in the enrichment score were selected to map the regulatory network of miR-98-5p and miR-669b-5p. The regulatory network of miR-98-5p contained 12 target genes and 5 transcription factors, of which the target gene *Hif1a* was also one of the enriched transcription factors (Figure 6B). For miR-669b-5p, the network contained eight target genes and the top five transcription factors (Figure 6C). *Runx2* and *Zfp219* are common transcription factors of miR-98-5p and miR-669b-5p targeted ADEGs.

### 3.5. In AngII-Treated MPC5 Cells, the Hydrogen Sulfide Pathway Is Dysregulated

**Figure 7:**
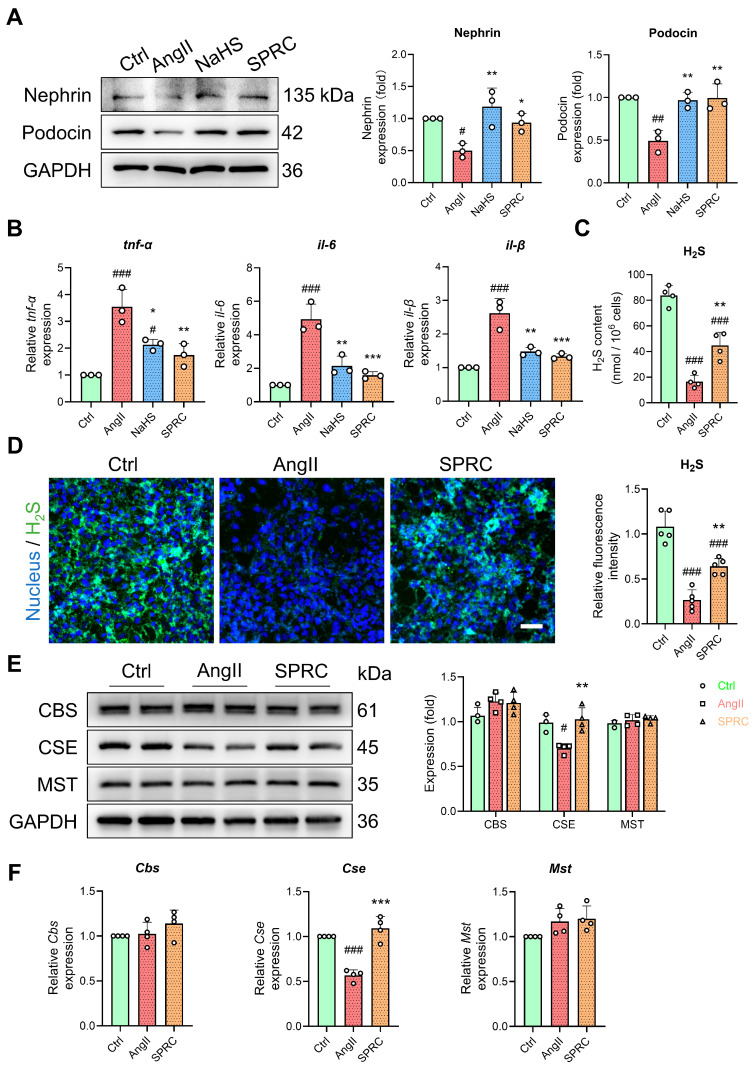
H_2_S pathway in AngII-treated MPC5 cells. (**A**) Western blot analysis of nephrin, podocin, and GAPDH in MPC5 cells under different stimuli. *n* = 3. (**B**) Normalized mRNA expression of Tnf-α, il-1β, and il-6. *n* = 3. (**C**) H_2_S content using spectrophotometer; *n* = 4. (**D**) H_2_S levels using fluorescent probe WSP-1. Bar = 50 μm; *n* = 5. (**E**) Western blot analysis of CBS, CSE, MST, and GAPDH in MPC5 cells under indicated stimuli; *n* = 3. (**F**) Normalized mRNA expression of *Cbs*, *Cse*, and *Mst*; *n* = 4. # *p* < 0.05, ## *p* < 0.01, ### *p* < 0.001 vs. AngII group, * *p* < 0.05, ** *p* < 0.01, *** *p* < 0.001 vs. Ctrl group.

Furthermore, we used mouse podocyte MPC5 cells to evaluate the therapeutic molecular mechanism of H_2_S donors in hypertensive nephropathy. AngII stimulation of MPC5 cells was used to mimic the pathological state of hypertensive nephropathy. AngII significantly reduced the podocyte-specific markers of nephrin and podocin (Figure 7A). In addition, we used NaHS and SPRC, a substrate donor for H_2_S synthesis, as endogenous and exogenous H_2_S generators [41,42]. NaHS and SPRC alleviated AngII-induced podocyte injury (Figure 7A). Similar to hypertensive renal injury in vivo, AngII significantly increased the transcription levels of intracellular *Tnf-α*, *il-1β*, and *il-6*, while NaHS and SPRC alleviated this inflammatory state (Figure 7B). This indicated that AngII puts cells in a state of damage and inflammation. Moreover, we also examined changes in H_2_S levels and synthases in this cellular state. The H_2_S content and fluorescent probe intensity in the MPC5 cells were significantly reduced under AngII stimulation, while SPRC partially restored the H_2_S level (Figure 7C,D). Furthermore, we monitored the changes in H_2_S synthase CBS, CSE, and MST. In the MPC5 cells stimulated by AngII, CSE was significantly downregulated, while the other two enzymes, CBS and MST, did not change (Figure 7E,F). This implicates the dysregulation of H_2_S pathway in AngII-treated MPC5 cells.

### 3.6. Changes in miR-98/669b, Related ADEGs, and Transcription Factors Were Observed in the Autophagy Regulatory Network

**Figure 8:**
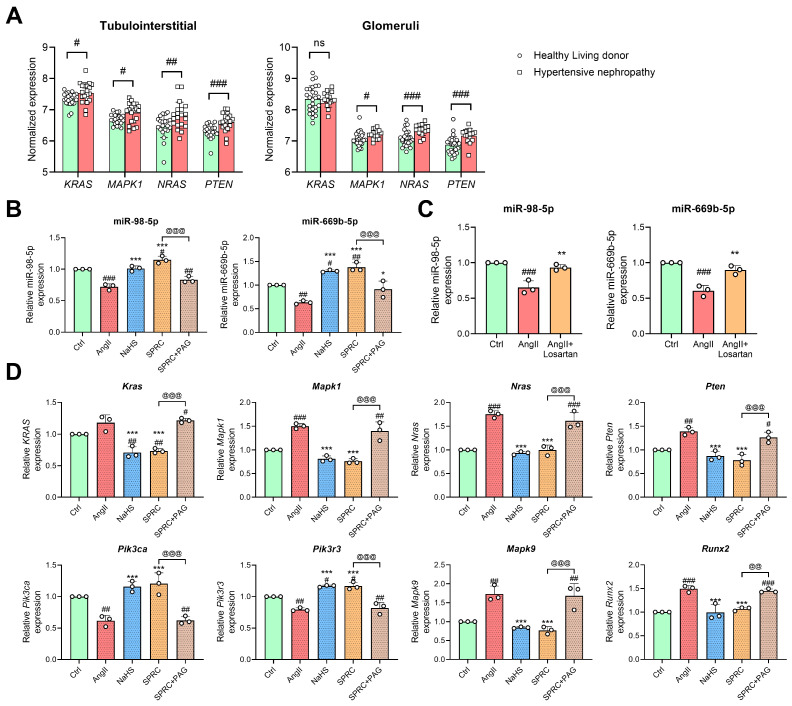
Validation of co-regulated DEMs, ADEGs, and TFs in hypertensive nephropathy clinical samples and AngII-treated MPC5 cells. (**A**) Normalized mRNA expression of *KRAS*, *MAPK1*, *NRAS*, and *PTEN* in the tubulointerstitial region (left) and glomeruli (right) of human renal biopsies. Data were extracted from GSE37455 and GSE37460. # *p* < 0.05, ## *p* < 0.01, ### *p* < 0.001 vs. healthy living donor group using two-sided unpaired *t*-test; *n* = 21 and 20 (**left**) or 27 and 15 (**right**) in the healthy and hypertensive group. (**B**,**C**) RNA expression of miR-98-5p and miR-669b-5p were measured in MPC5 cells by qPCR. (**D**) The mRNA levels of *Kras*, *Mapk1*, *Nras*, *Pten*, *Pik3ca*, *Pik3r3*, *Mapk9*, and *Runx2* in MPC5 cells were calculated using qPCR. For (**B**,**D**), the MPC5 cells were pre-incubated with or without 2mM PAG for 30 min and then pre-treated with NaHS (50 μM) or SPRC (50 μM) for 1 h before exposure to AngII (1 μM) treatment. NaHS, SPRC, and SPRC + PAG groups were all treated with AngII. # *p* < 0.05, ## *p* < 0.01, ### *p* < 0.001 vs. Ctrl group, * *p* < 0.05, ** *p* < 0.01, *** *p* < 0.001 vs. AngII group, and @@ *p* < 0.01, @@@ *p* < 0.001 vs. SPRC group. For (**C**), MPC5 cells were pre-stimulated with or without losartan (300 μM) for 12 h and then co-incubated with or without AngII (1 μM). ### *p* < 0.001 vs. Ctrl group, ** *p* < 0.01 vs. AngII group. One-way ANOVA coupled with Tukey’s multiple comparison post hoc test was used. Data are shown as mean ± SD, *n* = 3. ns, not significant.

To validate the reliability of the regulatory network, the co-regulated DEMs, ADEGs, and TFs were further verified in human hypertensive nephropathy renal samples and AngII-treated MPC5 cells. Co-regulated gene expression data were extracted from datasets GSE37455 and GSE37460 as previously described. The expression of *KRAS*, *MAPK1*, *NRAS*, and *PTEN* was significantly upregulated in the tubulointerstitial region of patients with hypertensive nephropathy compared with healthy living donors (Figure 8A, left panel). Similarly, the expression of *MAPK1*, *NRAS*, and *PTEN* was also significantly upregulated in the glomeruli of patients with hypertensive nephropathy (Figure 8A, right panel). Next, we detected co-regulated miRNAs and mRNAs by AngII and H_2_S donors in MPC5 cells using qPCR. In addition, PAG, an inhibitor of the CSE enzyme, was used to block SPRC for verification [37,43]. Similar to the results of the miRNA dataset, the AngII treatment decreased the expression levels of miR-98-5p and miR-669b-5p, but both endogenous and exogenous H_2_S donors reversed these changes in MPC5 cells (Figure 8B). To confirm the reliability of AngII’s regulation of these miRNAs, losartan was used here to bind to angiotensin receptors to block the effect of AngII. As shown in Figure 8C, losartan almost restored the expression of miR-98-5p and miR-669b-5p to the control level.

Moreover, the RNA expression of miRNA hub target genes and TFs were further detected. In the MPC5 cells, *Mapk1*, *Nras*, *Pten*, *Mapk9*, and *Runx2* were upregulated by AngII and downregulated by the H_2_S donors. In addition to the changes in the above genes, *Pik3ca* and *Pik3r3* were downregulated by AngII, which could be reversed by H_2_S donor treatment (Figure 8D). For the expression of *Kras*, AngII treatment increased its expression, with no statistical significance. However, both endogenous and exogenous hydrogen sulfide caused it to decrease. In addition, the regulatory role of SPRC on DEMs, ADEGs, and TFs was partially inhibited by PAG (Figure 8B,D).

**Figure 9:**
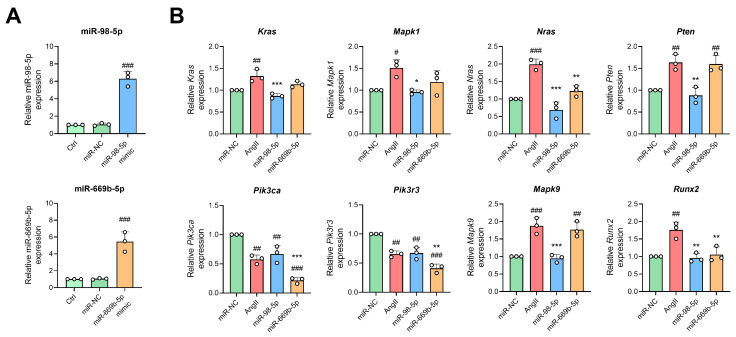
Overexpression of miR-98 and miR-669b. (**A**) RNA expression of miR-98-5p and miR-669b-5p were measured in MPC5 cells by qPCR. (**B**) The mRNA levels of *Kras*, *Mapk1*, *Nras*, *Pten*, *Pik3ca*, *Pik3r3*, *Mapk9*, and *Runx2* in MPC5 were calculated using qPCR. MPC5 cells were transfected with miR-NC, miR-98-5p mimic, and miR-669b-5p mimic. Forty-eight hours after infection, cells were incubated with or without AngII (1 μM) to detect RNA expression. Both miR-98-5p and miR-669b-5p mimic groups were incubated with AngII. # *p* < 0.05, ## *p* < 0.01, ### *p* < 0.001 vs. miR-NC group, * *p* < 0.05, ** *p* < 0.01, *** *p* < 0.001 vs. AngII group. One-way ANOVA coupled with Tukey’s multiple-comparison post hoc test was used. Data are shown as mean ± SD, *n* = 3.

Furthermore, miRNAs mimics were used to overexpress miR-98-5p and miR-669b-5p to find their genuine targets in the presence of AngII. The miR-98-5p mimic significantly increased the expression of miR-98-5p by about 6-fold, and the miR-669b-5p mimic increased the expression of miR-669b-5p by 5-fold, indicating successful overexpression (Figure 9A). In the case of co-treatment with AngII, the miR-98-5p mimic significantly reduced AngII-induced elevations in *Kras*, *Mapk1*, *Nras*, *Pten*, *Mapk9*, and *Runx2*, and the miR-669b-5p mimic decreased the expression of *Nras*, *Pik3ca*, *Pik3r3*, and *Runx2* changed by AngII (Figure 9B).

### 3.7. H_2_S Donors and miR-98/669b Overexpression Reversed Target Gene Changes, Restored Autophagy Flux, and Reduced Cell Damage

**Figure 10:**
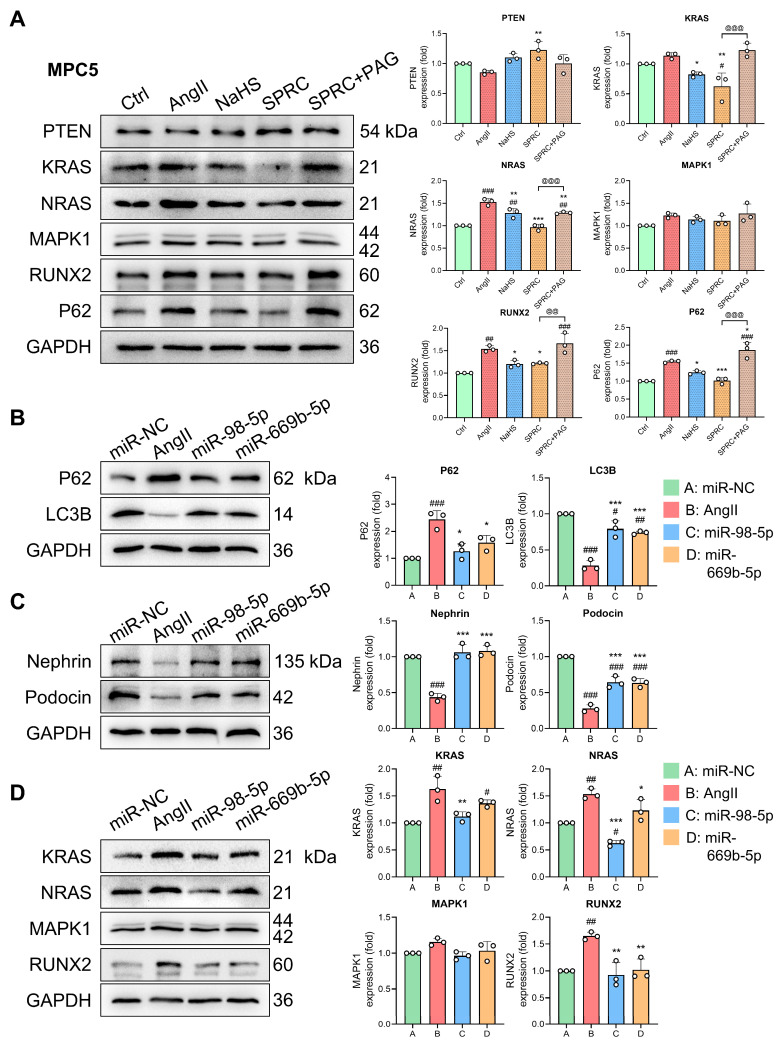
Validation of co-regulated DEMs, ADEGs, and TFs in protein levels. (**A**) Western blot analysis of PTEN, KRAS, NRAS, MAPK1, RUNX2, P62, and GAPDH protein expression levels in MPC5 cells under different stimuli. Cells were pre-incubated with or without PAG and then pre-treated with NaHS or SPRC for 1 h before exposure to AngII treatment. NaHS, SPRC, and SPRC + PAG groups were all treated with AngII. Expression levels were calculated by densitometric analysis and normalized to GAPDH. Protein quantification is shown in bar charts. # *p* < 0.05, ## *p* < 0.01, ### *p* < 0.001 vs. Ctrl group, * *p* < 0.05, ** *p* < 0.01, *** *p* < 0.001 vs. AngII group, and @@ *p* < 0.01, @@@ *p* < 0.001 vs. SPRC group. For (**B**–**D**), MPC5 cells were transfected with miR-NC, miR-98-5p mimic, and miR-669b-5p mimic and then incubated with AngII to detect the expression of autophagy markers P62 and LC3B (**B**), renal injury markers nephrin and podocin (**C**), and hub targets such as KRAS, NRAS, and MAPK1 as well as the TF RUNX2 (**D**) by Western blot analysis. Both miR-98-5p and miR-669b-5p mimic groups were incubated with AngII. With GAPDH as a normalized reference, protein quantification is shown in bar charts. Dark green, red, blue, and orange represent miR-NC, AngII, miR-98-5p, and miR-669b-5p mimic groups, respectively. # *p* < 0.05, ## *p* < 0.01, ### *p* < 0.001 vs. miR-NC group, * *p* < 0.05, ** *p* < 0.01, *** *p* < 0.001 vs. AngII group. One-way ANOVA coupled with Tukey’s multiple comparison post hoc test was used. Data are shown as mean ± SD, *n* = 3.

To further investigate the protein-level changes in this regulatory network, we investigated the protein expression of these co-regulated DEMs and TFs in MPC5 cells. As shown in Figure 10A, AngII stimulation significantly increased the expression of autophagy substrate P62 (SQSTM1) in the MPC5 cells, which implies a decrease in autophagic flux [44]. However, both the exogenous and endogenous hydrogen sulfide donors NaHS and SPRC restored the P62 expression to some extent. At the same time, similar to the gene-level changes, AngII significantly increased the levels of NRAS and RUNX2 in the MPC5 cells, and these changes were reversed by NaHS and SPRC. Furthermore, the alterations in KRAS, NRAS, RUNX2, and P62 induced by SPRC were partially offset by PAG (Figure 10A). In addition, we found small changes in PTEN and MAPK1, but most of them were not statistically significant. Subsequently, miRNA mimics were used to overexpress miR-98-5p and miR-669b-5p in the presence or absence of AngII to detect changes in autophagy markers and kidney injury markers. The AngII stimulation significantly increased P62 and decreased LC3B expression, implying a decrease in autophagy flux. However, the overexpression of miR-98-5p and miR-669b-5p significantly restored the expression of P62 and LC3B and improved intracellular autophagy (Figure 10B). In terms of kidney injury indicators, AngII reduced the expression of the podocyte markers nephrin and podocin, but the overexpression of miR-98-5p and miR-669b-5p restored the AngII-induced reduction in these two proteins (Figure 10C). Finally, WB analysis was conducted to check which hub target genes and TFs were altered in response to miR-98-5p and miR-669b-5p overexpression as well as AngII stimulation. As shown in Figure 10D, the miR-98-5p mimic reduced the AngII-induced increases in KRAS, NRAS, and RUNX2. Meanwhile, the miR-669b-5p mimic partially restored the AngII-induced changes in NRAS and RUNX2.

Collectively, in the hypertensive nephropathy samples and AngII-induced podocyte models, the renal injury markers were increased, autophagic flux was decreased, and differential miRNAs (miR-98-5p and miR-669b-5p), hub target genes (*KRAS* and *NRAS*), and transcription factor (*RUNX2*) were dysregulated. The endogenous and exogenous H_2_S donors restored the expression of miR-98-5p and miR-669b-5p as well as other hub genes in the regulatory network. The overexpression of miR-98-5p and miR-669b-5p increased autophagic flux, reduced markers of kidney injury, and also restored the abnormal expression of KRAS, NRAS, and RUNX2. This may be the mechanism by which H_2_S donors alleviate hypertensive nephropathy.

## 4. Discussion

Hypertensive nephropathy is one of the major causes of chronic kidney disease and end-stage renal disease [45]. Recently, emerging evidence has shown that autophagy plays a crucial role in the maintenance of kidney function and kidney diseases [46,47]. Targeting the autophagy pathway may have therapeutic benefits in the treatment of kidney diseases [48]. In acute kidney injury, autophagy is induced in the proximal tubule as a protective mechanism [47]. The deletion of key autophagy proteins increases P62 levels and oxidative stress, which impairs renal function [49]. In diabetic nephropathy, the dysregulation of autophagy in podocytes, glomerular endothelial cells, and proximal tubules is thought to contribute to disease progression [50]. Dysregulated autophagy has also been reported in many kidney diseases, such as tubulointerstitial fibrosis, focal segmental glomerulosclerosis, and polycystic kidney disease [48,51]. However, the role of autophagy in hypertensive nephropathy or kidney injury caused by hypertension has not yet been elucidated. Here, we have demonstrated that autophagy may be involved in AngII-induced hypertensive nephropathy. We screened out 109 differentially expressed miRNAs and the corresponding 4667 target genes after AngII treatment. Then, 21 of the 233 hub genes, selected from the top 5% CytoHubba algorithm, were related to autophagy. If hub genes are not counted, the 57 of the 4667 target genes were also associated with autophagy. This indicates that autophagy-related genes accounted for a large proportion of the hub target genes of DEMs after the AngII treatment.

Studies have shown that hydrogen sulfide play a therapeutic role by regulating the autophagy pathway [52]. In renal disease, exogenous H_2_S treatment can protect the kidney from ROS-induced autophagy and renal injury by inhibiting the ROS-AMPK pathway [53]. However, there are few reports on the relationship between hydrogen sulfide and autophagy in hypertensive nephropathy. Our study has demonstrated that hydrogen sulfide donor treatment may affect the autophagy pathway altered by AngII. We identified 70 DEMs and the corresponding 2828 target genes in the AngII_H_2_S vs. AngII group, 44 of which were associated with autophagy, and 13 of the 141 key target genes were associated with autophagy. To our knowledge, this is the first bioinformatic report describing the regulation of autophagy pathways by hydrogen sulfide donors in hypertensive nephropathy.

miRNA dysregulation is an important component of epigenetic regulation, and studies have identified a large number of miRNAs that play a role in hypertensive nephropathy [54], and the role of hydrogen sulfide and microRNA crosstalk has also gradually emerged in kidney diseases [25,55]. In the present study, 12 miRNAs targeting key ADEGs were altered upon AngII treatment, and correspondingly, 11 miRNAs were altered upon co-treatment with AngII and hydrogen sulfide. Among them, miR-98-5p and miR-669b-5p were co-altered miRNAs, and AngII caused their decrease, while the hydrogen sulfide donors significantly increased the expression of both. Studies have shown that miR-98-5p is decreased in the kidney tissue of diabetic mice and in high-glucose-treated human podocytes (HPCs) and HK2 cells, and the overexpression of miR-98-5p inhibits apoptosis, proteinuria, and the renal fibrosis process [56,57]. In cardiovascular diseases, the overexpression of miR-98-5p can also protect cardiomyocytes from apoptosis [58,59]. These all indicate the protective effect of miR-98-5p. For miR-669b-5p, it was only found in mus musculus, and there are few reports on its function. But for its family, miR-669c was protective in a mouse model of ischemic stroke, and long-term miR-669a treatment attenuated chronic dilated cardiomyopathy in dystrophic mice [60,61]. These studies have suggested a possible protective role of the miR-669 family in cardiovascular diseases.

This study focused on changes in miR-98-5p and miR-669b-5p, but the remaining DEMs may also play a role in the progression of hypertensive nephropathy. Among the other AngII-upregulated miRNAs, previous studies have shown that silencing microRNA-132 reduced renal fibrosis by selectively inhibiting myofibroblast proliferation [62]. The miR-132 family also directly targets the pro-autophagy FoxO3 transcription factor [63]. miR-762 is significantly upregulated in septic acute kidney injury [64]. For other miRNAs downregulated by AngII, the most studied is miR-204-5p. miR-204-5p is downregulated in renal biopsy specimens from patients with hypertensive nephrosclerosis, diabetic nephropathy, and in the kidneys of rats fed with a high-salt diet [65,66]. Furthermore, the knockdown or knockout of miR-204-5p results in substantial pathological changes in the kidneys as well as proteinuria [66], and the protective role of miR-204 in chronic kidney disease is related to SHP2 and STAT3 [67]. For the downregulated DEMs in the hydrogen sulfide group, miRNA-363-3p promotes apoptosis in response to heavy-metal-induced kidney injury by downregulating phosphoinositide 3-kinase expression [68]. In H_2_S-upregulated DEMs, miR-129-5p attenuates LPS-induced podocyte injury and acute kidney injury, and this kind of protection is related to the regulation of the NF-κB signaling pathway [69]. In short, these altered miRNAs may be the underlying mechanism behind AngII-induced hypertensive nephropathy and the therapeutic effect of hydrogen sulfide.

A total of 21 and 13 ADEGs were screened out, and their potential biological functions were identified by GO and KEGG enrichment analysis. Interestingly, these ADEGs collectively affect protein phosphorylation, phosphatidylinositol 3-kinase complex, protein binding, and protein kinase activity. These aspects are all related to autophagy. Protein kinases are integral to autophagy. Both autophagy initiation and autophagy signaling utilize kinase mechanisms [70]. Among them, kinase-catalyzed phosphorylation is an important part of the most-studied post-translational modification of autophagy [71]. For example, phosphatidylinositol 3-kinase (PI3K) interacts with various regulatory proteins to form multiple complexes that selectively participate in different stages of autophagy. A complex of PI3K and ATG14 is involved in the formation of autophagic vesicles [72]. In addition, the ADEGs that affect these biological functions may be genes such as PTEN, AMPK1, KRAS, and NRAS. In the enrichment study of signaling pathways, autophagy and FoxO signal transduction are commonly enriched signaling pathways. This further illustrates that AngII and H_2_S donors affect the autophagy pathway in the kidney. The role of the FoxO transcription factor family in autophagy has been widely reported. For example, FoxO1 and FoxO3 enhance autophagic flux by increasing the expression of autophagy genes, such as Ulk2, Becn1, Lc3b, Atg12, and GabarapL1 [73]. Furthermore, SNAI2 is transcriptionally activated by FoxO3 and interacts with FoxO3 to form a feed-forward regulatory loop to enhance the expression of autophagy genes [74]. In addition to general autophagy, FoxO3 is also involved in the regulation of mitophagy [75]. Among the other AngII-enriched signaling pathways, mTOR signal transduction is closely related to autophagy, and mTORC1 in the signaling pathway is also a promising pharmacological target for regulating autophagy [76]. Future experiments will be necessary to explore the potential biological functions of these ADEGs regulated by AngII and hydrogen sulfide donors.

After the PPI analysis, we validated co-regulated DEMs, ADEGs, and TFs using clinical patient databases as well as several cell experiments. In the kidney cell experiments, AngII stimulation significantly increased the expression of the autophagy substrate P62, implying a decrease in autophagic flux. Previous studies have shown that P62, a substrate of the autophagy–lysosomal degradation pathway, was observed to accumulate in the kidneys of STZ-induced diabetic mice, Wistar obese rat bodies, and kidney biopsies of patients with type 2 diabetes, suggesting that autophagy is impaired [77]. Targeting the autophagy pathway to activate and restore autophagic activity may be renoprotective. For example, the endogenous calpain inhibitor calpastatin can maintain autophagy to alleviate AngII-mediated podocyte injury [9]. In addition, AngII can also induce the activation of TLR4 to inhibit autophagy and induce apoptosis and kidney injury [78]. At the same time, changes in KRAS and NRAS were also observed. On the one hand, the inhibition of KRAS increases autophagic flux, and the inhibition of KRAS/RAF/MEK/ERK signaling triggers autophagy [79,80]. On the other hand, for NRAS, there are not many corresponding reports. In the MPC5 cells, we found that both endogenous and exogenous hydrogen sulfide reversed AngII-induced KRAS and NRAS changes to some extent, which may partially restore autophagy in cells. Changes in PTEN and MAPK1 were found in the clinical samples and kidney cells. Published findings suggest that PTEN activates autophagy through the AKT/mTOR pathway [81]. Furthermore, autophagic flux and ATG7 (autophagy-related 7) levels were increased by the activation of MAPK1/3 [82]. We found that PTEN and MAPK1 were increased at the gene level, which may be related to the positive feedback regulation of autophagy in the early stage of disease. In cervical cancer cells, the overexpression of miR-98-5p significantly increased the expression of the PTEN protein, and silencing miR-98-5p had the opposite result [83]. In the future, more experiments are needed to explore the effect of timing of AngII regulation on PTEN and autophagy.

The synergistic effect of transcription factors and miRNAs has not yet been fully elucidated, and this study is also the first attempt to reveal the interactions between DEMs, hub target genes, and transcription factors in the treatment of AngII-induced hypertensive kidney injury with hydrogen sulfide donors. We identified and validated one transcription factor, Runx2, that co-regulates miR-98-5p and miR-669b-5p target hub genes. Runx2 is a well-known transcription factor for skeletal development, but its role in non-bone tissues is gradually being reported. Studies have shown that AngII can significantly increase the level of Runx2 in aortic tissue and human aortic smooth muscle cells, which is similar to our validation results [84,85]. In addition, the activation of Runx2 promotes aortic valve fibrosis as well as aortic fibrosis and stiffness in patients with type 2 diabetes [86,87]. In chronic kidney disease, Runx2 is deacetylated by SIRT6 to inhibit osteogenic trans differentiation of vascular smooth muscle cells [88]. Runx2 is also regulated by miRNAs. The downregulation of miR-204 promoted hyperproliferation and calcified lesions of pulmonary artery smooth muscle cells by increasing the expression of the transcription factor Runx2 [89]. Increased miR-204-5p also suppressed Runx2 expression, thereby inhibiting aortic valve calcification [90]. Interestingly, studies have shown that Runx2 also promoted autophagy by enhancing the trafficking of LC3B vesicles [91]. In this study, the transcription factor Runx2 was upregulated by AngII, and this upregulation was reversed by endogenous and exogenous hydrogen sulfide donors. These results suggest that the transcription factor Runx2 may be a good therapeutic target for hydrogen sulfide donors to treat the pathological process of renal fibrosis in hypertensive nephropathy. The detailed functions and underlying mechanistic pathways of Runx2 require further investigation. More in vitro and in vivo studies and validations are needed in the future to elucidate the role of these new markers, including DEMs, hub ADEGs, and TFs in hypertensive nephropathy and hydrogen sulfide therapy.

## 5. Conclusions

By using bioinformatics analysis, 109 DEMs in mouse kidney tissue after treatment with AngII alone and 70 DEMs after treatment with a combined treatment of AngII and H_2_S donors were identified. Through the regulatory network and validation study, the H_2_S donor treatment increased autophagic flux, reduced renal cell injury, and restored the two identified and co-regulated miRNAs, miR-98-5p and miR-669b-5p, attenuated the dysregulation of ADEGs (*KRAS*, *NRAS*) and a key transcription factor (*RUNX2*). These findings help to define the pathophysiological mechanism of AngII-induced hypertensive nephropathy and shed new light on the therapeutic effects of hydrogen sulfide donors.

## Data Availability

All data generated or analyzed during this study are included in this published article, it’s supplementary information files, and publicly available repositories. The datasets GSE92857, GSE37455, and GSE37460 were downloaded from the GEO database.

## Supplementary Materials

The following supporting information can be downloaded at: https://www.mdpi.com/article/10.3390/antiox13080958/s1. Figure S1: Profiles of the non-coding RNA expression profile dataset GSE92857, Table S1: Autophagy-related gene set in mouse, Table S2: Primer sequences, Table S3: Differentially expressed miRNAs between AngII and Ctrl group, Table S4: Differentially expressed miRNAs between AngII_H_2_S and AngII group, Table S5: DEMs and their target ADEGs in AngII vs Ctrl and AngII_H_2_S vs AngII comparison.
