# Supplementary figures and images for: Identification and Validation of the miR/RAS/RUNX2 Autophagy Regulatory Network in AngII-Induced Hypertensive Nephropathy in MPC5 Cells Treated with Hydrogen Sulfide Donors
Source: Antioxidants (Basel). 2024 Aug 7;13(8):958. doi: 10.3390/antiox13080958 (PMC11351630; doi:10.3390/antiox13080958)

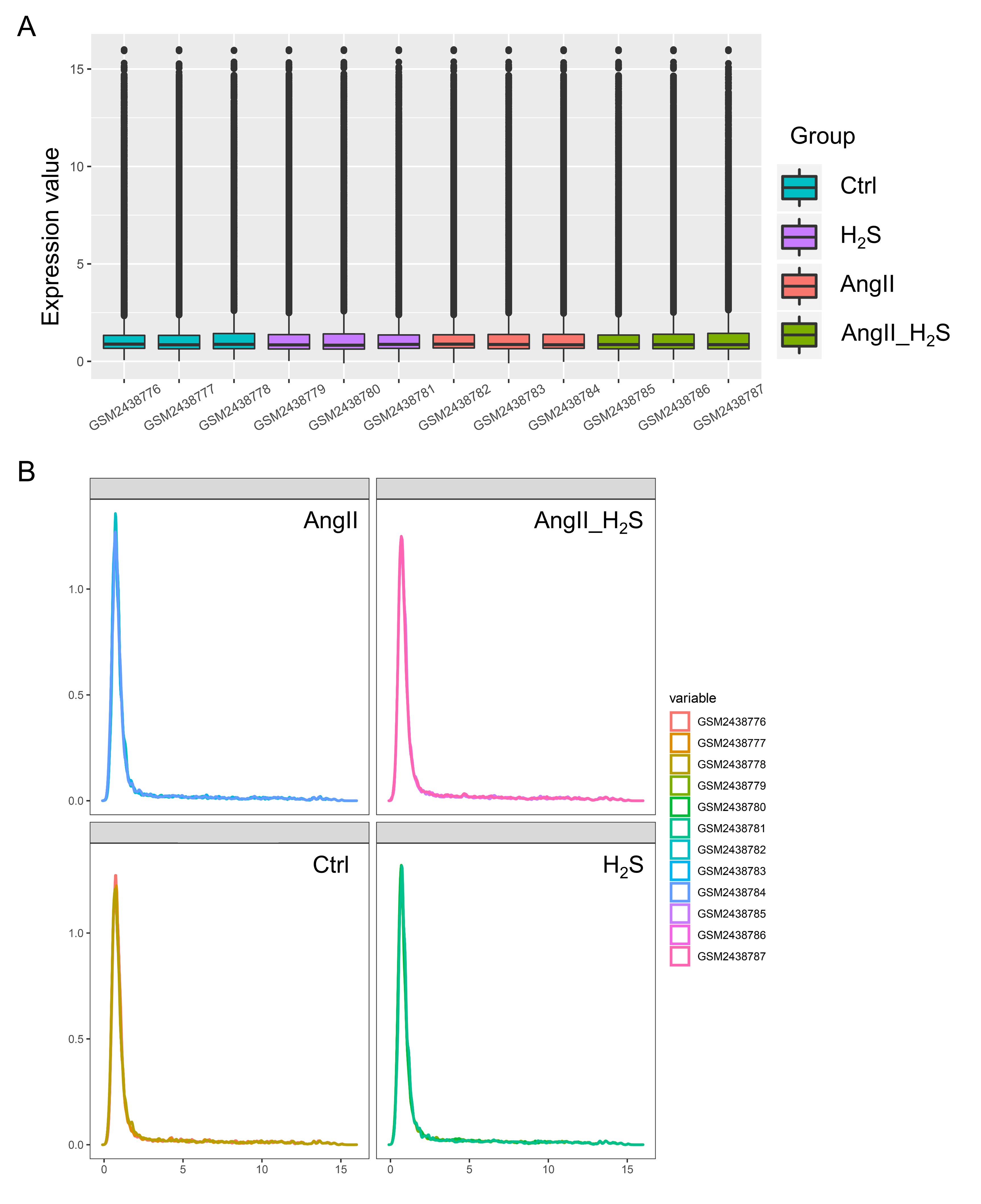

Supplement: Supplementary file 1 [file antioxidants-13-00958-s001.zip › Supplementary Figure S1.TIF]
